# Endemic penetrance of SARS-CoV-2 has impacted marginally on immunity to spike protein of human coronaviruses

**DOI:** 10.1038/s42003-025-09474-x

**Published:** 2026-01-03

**Authors:** Tara Lancaster, Gokhan Tut, Panagiota Sylla, David Bone, Christopher Bentley, Eliska Spalkova, Azar Jadir, Rachel Bruton, Katie Spencer, Soumyajit Mallick, Ahmed Elzaidi, Siobhan Plass, Nayandeep Kaur, Megan Butler, Samuel Hulme, Alexander C. Dowell, Maria Krutikov, Oliver Stirrup, Borscha Azmi, Andrew Hayward, Andrew Copas, Laura Shallcross, Paul Moss

**Affiliations:** 1 School of Infection, Inflammation and Immunology, College of Medicine and Health, University of Birmingham, Birmingham, UK; 2 UCL Institute of Health Informatics, London, UK; 3 UCL Institute for Global Health, London, UK; 4 Health Data Research, London, UK; 5 UK Health Security Agency, London, UK; 6 University Hospitals NHS Foundation Trust, Birmingham, UK

**Keywords:** Infectious diseases, Infectious diseases

## Abstract

SARS-CoV-2 has emerged as the 5th endemic coronavirus and immunological cross protection between coronaviruses will influence their infectivity and clinical impact. We determined adaptive immunity against the spike protein of each human coronavirus during the course of the COVID-19 pandemic. A characteristic pattern of HCoV immunodominance, dominated by OC43 and 229E, was apparent prior to SARS-CoV-2 and was largely unaffected by SARS-CoV-2 infection, which itself elicited moderate antibody titre. Vaccination or hybrid immunity elicited supraphysiological levels of coronavirus-specific antibodies, only a proportion of which was cross-reactive with SARS-CoV-2 spike indicating substantial backboosting of HCoV-specific responses. SARS-CoV-2 vaccination focused antibody responses against the S1 domain of SARS-CoV-2 spike whilst T cell responses recognised peptides equivalently across S1 and S2. Coronavirus-specific T cells exhibited strong production of IFN-γ, IL-2 and CXCL8. In summary, the entry of SARS-CoV-2 into its ecological niche has impacted marginally on relative immunity against other human coronaviruses although vaccination provides a modest antibody increment which is unlikely to be maintained. Further, although SARS-CoV-2 vaccination elicits spike-specific adaptive immune responses that are focused against the S1 domain, thereby favouring neutralising antibodies, the natural history of HCoV immunity indicates that adaptive responses may transition towards S2 recognition across the life course.

## Abstract (web summary)

A prospective analysis reveals that emergence of SARS-CoV-2 has had only a marginal impact on the immune response to spike protein of other endemic human coronaviruses and predicts limited cross protection against infection.

## Introduction

The emergence of SARS-CoV-2 led to the COVID-19 pandemic, which is estimated to have led to the death of over 7 million people. SARS-CoV-2 is now endemic within the human population and joins the betacoronaviruses OC43 and HKU1, and alphacoronaviruses NL63 and 229E, as the 5th endemic human coronavirus. These latter 4 viruses are typically seasonal in nature, cause respiratory tract infections, and have been traditionally termed as the human coronaviruses (HCoV)^1–3^.

The spike protein of the betacoronavirus and alphacoronavirus CoV genera shows variable degrees of sequence homology between viruses (Supplementary Fig. 1) and the nature of immunological cross-reactivity will impact substantially on HCoV infectivity and clinical significance. The fact that the 5 viruses co-exist indicates that sterilising immunity between viruses has not been established and each virus has acquired an ecological niche. Nevertheless, cross-reactive antibody and T cell responses have been observed against a range of viral proteins.

The clinical importance of cross-reactive coronavirus-specific immune responses remains unclear^4^. T cell responses against SARS-CoV-2 have been demonstrated within pre-pandemic blood samples^5–7^, and IgG responses against the spike S2 domain have also been observed, most notably in childhood^8^. The asynchronous seasonal incidence and rare co-infection profile of HCoV indicates that niche segregation due to immune competition may have been established^1^. Despite this, there is relatively little evidence to suggest that a history of prior HCoV infection is protective against COVID-19, although a recent HCoV infection may potentially provide some benefit^9^. In contrast, pre-existing immunity against HCoV might actually be detrimental to protection from SARS-CoV-2 through preferential boosting of pre-existing HCoV-specific B or T cell clones with low affinity against SARS-CoV-2 rather than generation of primary high-affinity clones^10–13^.

In common with other HCoV, SARS-CoV-2 has established a clinical pattern of repeated infection and remains a considerable health concern, most notably for the elderly and those with immune suppression. As such, vaccination programmes and other approaches to limit morbidity are likely to remain as major health priorities. Given this background, it is now critical to characterise the interrelationship between SARS-CoV-2 and HCoV-specific immune responses^14^. Whilst some degree of humoral immune boosting against HCoV has been observed following SARS-CoV-2, details on the magnitude and specificity of humoral and cellular immunity following infection and/or vaccination are incomplete.

Spike protein is the primary determinant of viral infectivity, the major target of neutralising antibodies, and the antigen used in most vaccines. Here, we analysed SARS-CoV-2 and HCoV spike-specific antibody and cellular immune responses during the course of the SARS-CoV-2 pandemic. Given the importance of age as a primary determinant of COVID-19 risk, this work was undertaken across the adult life course in the setting of long-term care facilities. Our findings reveal that HCoV-specific immune responses have a defined profile of immunodominance that has not been disrupted through the introduction of SARS-CoV-2. SARS-CoV-2 infection and vaccination enhance HCoV-specific antibodies both through back boosting of HCoV-specific clones and a component of predominantly S2-specific cross-reactive antibodies. Much less impact of vaccination is observed on HCoV-specific T cell responses, which are largely targeted against S2. These findings are important for understanding the potential impact of SARS-CoV-2 evolution on the clinical burden of HCoV and offer insight into how the specificity of SARS-CoV-2-specific immune responses may evolve over time.

## Methods

### Study design

The VIVALDI study (ISRCTN14447421) is a prospective cohort study which was set up in May 2020. The study investigated SARS-CoV-2 transmission, infection outcomes and immunity in both residents and staff of LTCFs in England that provide residential and/or nursing care for adults aged 65 years and over^15,16^. Ethical permission was provided by South Central-Hampshire B Research Ethics Committee, reference 20/SC/0238. All ethical regulations relevant to human research participants were followed.

Eligible LTCFs were identified by the Care Provider’s Senior Management Team, or by the National Institute for Health Research (NIHR) Clinical Research Network. Pseudonymised clinical (vaccination status, PCR test results, hospitalisation, death) and demographic (age, sex, staff member versus resident) data were retrieved for staff and residents from participating LTCFs through national surveillance systems. Written informed consent was provided by all participants for blood sample collection, or if residents lacked the capacity to consent, a personal or nominated consultee was identified to act on their behalf.

### Sample collection and preparation

Blood sampling for the pre-vaccination cohort was carried out from 23rd June 2020 until December 2020 as previously described^17^. Blood sampling was carried out from 21st July 2022 until 21st of February 2023 for the post-vaccination cohort, and donors had received 2+ vaccine doses. Blood samples collected in sodium heparin tubes were sent to the University of Birmingham, and a serum tube was also obtained for The Doctors Laboratory, where anti-nucleocapsid IgG (N) testing using the Abbott immunoassay was performed. Ethical approval for this study was obtained from the South Central—Hampshire B Research Ethics Committee, REC Ref: 20/SC/0238.

### Data linkage

Abbott antibody test results were submitted to the COVID-19 datastore (https://data.england.nhs.uk/covid-19/), pseudonymised and linked to routinely held data on age, sex, LTCF, role (staff or resident), and results of PCR or lateral flow device (LFD) SARS-CoV-2 testing performed through the national SARS-CoV-2 testing programme.

This data was linked to vaccination status (date and vaccine type) derived from the National Immunisations Management System and dates and diagnostic codes for hospitalisations recorded in the Hospital Episode Statistics (HES) dataset, as well as for any deaths from the Office for National Statistics (ONS) dataset. Individual-level records were further linked to each LTCF using the unique Care Quality Commission location ID (CQC-ID), allocated by the Care Quality Commission, who regulate all providers of health and social care in the UK.

### Inclusion criteria

LTCF Staff and residents were eligible for inclusion if samples could be linked to a pseudo-identifier to enable linkage to vaccination records. We included samples from participants that had received a primary vaccine course with or without a third, fourth and fifth dose. Participants sampled in the 10 days following the last vaccine administration were excluded from further analysis to ensure peak immune responses were reached after each vaccine prior to sampling. Due to limited PCR testing in the first wave of the pandemic and after March 2022, it was not possible to determine when individuals had been infected with SARS-CoV-2 based on PCR alone. Prior infection with SARS-CoV-2 was defined based using the MSD platform, where a nucleocapsid-specific value of 1200 AU/ml or above was taken as evidence of prior infection. No statistical methods were used to pre-determine sample sizes, but our sample sizes are similar to those reported in previous publications^18^.

### Sample preparation

Samples were processed within 24 h of reception at the University of Birmingham. Blood was spun at 300 x *g* for 5 min. Plasma was removed and spun at 500 × *g* for 10 min prior to storage at −80 °C. Remaining blood was separated using a SepMate (Stemcell) density centrifugation tube. The resulting PBMC layer was washed twice with RPMI and rested overnight in R10 (RPMI + 10% FBS + Pen/Strep) media at 37 °C in 5% CO2.

### Serological analysis of coronavirus-specific immune response

Quantitative IgG antibody titres were measured against trimeric ancestral SARS-CoV-2 spike (S) protein, SARS-CoV-2 Nucleocapsid (N) protein and spike proteins from endemic human coronaviruses (OC43, HKU1, NL63 and 229E) using the MSD V-PLEX COVID-19 Coronavirus Panel 2 (IgG) Kit following manufacturer’s instructions (Lot number K0081806) (Supplementary Data 1). Briefly, 96-well plates were blocked. Following washing, plasma samples were diluted at 1:5000 in diluent and added to the wells with the reference standard and internal controls. After incubation, plates were washed, and SULFO-tag anti-IgG detection antibodies were added. Plates were washed and immediately read using a MESO TM QuickPlex SQ 120 system. Data were generated by Methodical Mind software and analysed with MSD Discovery Workbench (v4.0) software. Presented data were adjusted for sample dilutions.

### Cross-reactivity assay

Assay was carried out as previously described^19^. Donor plasma was diluted 1:10 in PBS prior to incubation at 37 °C for 30 min with an equal volume of either recombinant SARS-CoV-2 S1 protein (R&D Systems, 10569-CV-100) or recombinant SARS-CoV-2 S2 protein (R&D Systems, 10594-CV-100) at a concentration of 500 µg/ml in PBS, or with PBS alone. Plasma was then diluted at 1:5000 in diluent and assayed using an MSD V-PLEX COVID-19 Coronavirus Panel 2 (IgG) Kit (Lot number K0081806) as above.

### Quantification of coronavirus-specific cellular responses by FluoroSpot

Pepmix pools containing 15-mer peptides overlapping by 11aa from either the S1 or S2 protein domains of SARS-CoV-2 (P0DTC2) and the four endemic human coronaviruses (OC43, HKU1, NL63 and 229E) were purchased from JPT Peptide Technologies (Germany). T cell responses of post-vaccination samples to the above peptide mixes were determined using a Human IFNγ/IL-2 FluoroSpot Plus Kit (Mabtech, Sweden). 2−3 × 10⁵ isolated PBMC rested overnight in R10 (RPMI + 10% FBS + Pen/Strep) were seeded in duplicate with co-stimulus anti-CD28 (CD28-A, 1:1000), and stimulated with peptide mixes at 1 μg/ml per peptide, anti-CD3 (CD3-2) as a positive control, or DMSO as a negative control. After 16–18 h, the supernatants were harvested and stored at −80 °C, whilst ELISpot plates were read using the AID iSpot FluoroSpot reader. Mean spot counts in DMSO-treated negative control wells were deducted from the mean of test wells to generate normalised spot counts for all other treated wells. Cut-off values were previously determined^20^.

### Quantification and analysis of cytokine production

Cytokine concentrations within Fluorospot supernatants and donor plasma were assayed using a LEGENDplex™ COVID-19 Cytokine Storm Panel 1 (BioLegend, Lot Number B377365) according to the manufacturer’s instructions. Data were analysed using the LEGENDplex™ Data Analysis Software Suite (BioLegend).

### Statistics and reproducibility

Sample size and replicates are shown in each figure. The ranges of sample sizes were between 24 and 152 in relation to infection and/or vaccination status (Table 1). Adsorption data was performed on 18 and 23 donors in relation to vaccination status. Serological analysis on the MSD platform was done in triplicate with individual ELISpot assay. All data were checked for normality using the Kolmogorov–Smirnov test, and for comparative analysis of two groups, a Mann–Whitney test was applied. A Friedman test was applied for non-parametric paired data. For comparative analysis with three or more groups, a Kruskal–Wallis test was used. For multiple comparisons of non-parametric data, an uncorrected Dunn’s test was used. Spearman’s rank correlation coefficients (*r*) were calculated and tested for correlations. *P*-values < 0.05 were considered to be statistically significant.

**Table 1:** Donor demographics

	Uninfected unvaccinated (*n* = 124) Pre-SARS-CoV-2	Infected unvaccinated (*n* = 152) Infection	Uninfected vaccinated (*n* = 24) Vaccination	Infected and vaccinated (*n* = 69) Hybrid immunity
**Median vaccines received**	Nil	Nil	4	3
**Median age (IQR)**	64 (57–86)	63 (53–82)	61 (52–85)	60 (48–88)
**Median days between sample and latest vaccine (IQR)**	N/A	N/A	135 (43–316)	133 (101–314)
**Female**	92 (74%)	119 (78%)	17 (71%)	55 (80%)
**Male**	32 (26%)	33 (22%)	7 (29%)	14 (20%)
**Primary course BNT162b2 (Pfizer-BioNTech)**	N/A	N/A	14 (58%)	38 (55%)
**Primary course ChAdOx1 (Oxford AstraZeneca)**	N/A	N/A	10 (42%)	31 (45%)

### Reporting summary

Further information on research design is available in the Nature Portfolio Reporting Summary linked to this article.

## Results

### Robust humoral immunity against HCoV is seen across the lifecourse and is boosted by SARS-CoV-2 infection or vaccination

Blood samples were obtained from staff and residents within LTCFs and split into four cohorts on the basis of prior infection and/or vaccination with SARS-CoV-2: uninfected/unvaccinated (*n* = 124), uninfected/vaccinated (*n* = 24), infected/unvaccinated (*n* = 152), and infected/vaccinated (*n* = 69) (Table 1; Supplementary Data 1). Primary series vaccination was undertaken with BNT162b2 (Pfizer-BioNtech) or ChAdOx1 vaccines and booster vaccination with BNT162b2.

**Fig. 1:**
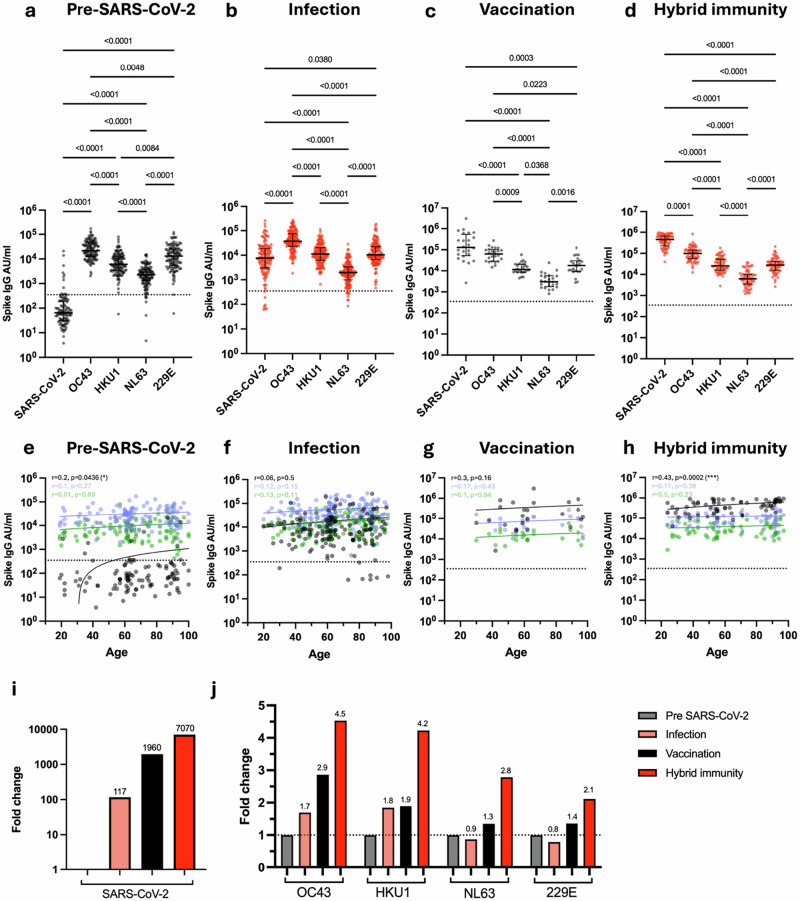
Antibody titre against spike from SARS-CoV-2 and HCoV in relation to SARS-CoV-2 infection and vaccination status. Spike-specific IgG titre against SARS-CoV-2, OC43, HKU1, NL63 and 229E in SARS-CoV-2-seronegative unvaccinated donors (**a**, *n* = 124), SARS-CoV-2-seropositive unvaccinated donors (**b**, *n* = 152), SARS-CoV-2-seronegative vaccinated donors (**c**, *n* = 24) and SARS-CoV-2-seropositive vaccinated donors (**d**, *n* = 69). Kruskal–Wallis (Dunn’s multiple comparisons test). Black lines indicate median titre and interquartile range, dotted black line indicates the cutoff. Correlation of age with Spike-specific IgG titre against SARS-CoV-2 (black), OC43 (blue) and HKU1 (green) in seronegative unvaccinated donors (**e**, *n* = 124), seropositive unvaccinated donors (**f**, *n* = 152), seronegative vaccinated donors (**g**, *n* = 24) and seropositive vaccinated donors (**h**, *n* = 69). The Spearman correlation coefficient (*r*) and *P*-values are shown. Fitted lines are linear regressions. No age-related correlations were observed with HCoVs (Supplementary Fig. 2). **i** Fold change in median SARS-CoV-2 spike IgG titre in seropositive unvaccinated (light red), seronegative vaccinated (black) and seropositive vaccinated (red) donors in relation to seronegative unvaccinated donors. Fold changes are listed. **j** Fold change in median OC43, HKU1, NL63 and 229E spike-specific IgG in seropositive unvaccinated (light red), seronegative vaccinated (black) and seropositive vaccinated (red) donors in relation to seronegative unvaccinated donors (grey). Fold changes are listed, black dotted line denotes a fold change of 1. Source data: Supplementary Data 1.

Initial studies focused on IgG titre against spike protein from SARS-CoV-2 and the four endemic coronaviruses (OC43, HKU1, NL63, 229E) (Fig. 1).

As expected, SARS-CoV-2 uninfected and unvaccinated donors exhibited low levels of SARS-CoV-2 spike IgG antibodies, although positive values were observed in a few cases (Fig. 1a), most notably in older donors and potentially indicating an increase in spike protein cross-reactivity with age (Fig. 1e, *p* = 0.04). High baseline levels of spike-specific antibody towards the four endemic coronaviruses were observed in all donors, with OC43 and 229E being particularly immunodominant.

The increment in spike-specific IgG titre against each virus after SARS-CoV-2 infection or vaccination was next assessed by relative fold-change increase, calculated as the median AU/ml values within the ‘Infection’, ‘Vaccine’ and ‘Hybrid Immunity’ groups divided by the median AU/ml value within the ‘Pre-SARS-CoV-2’ group. SARS-CoV-2 infection boosted SARS-CoV-2-specific spike IgG titre by 117-fold (Fig. 1b, i) together with a 1.7-fold and 1.8-fold increment against betacoronavirus OC43 and HKU1 spike protein (Fig. 1j) but no increase against alphacoronaviruses. Of note, SARS-CoV-2-specific antibody titre remained lower than that seen against OC43 or 229E.

In comparison to infection, antibody responses against all coronaviruses were increased much more markedly in vaccinees, with fold-titre increases of 1960, 2.9, 1.9, 1.3 and 1.4 against SARS-CoV-2, OC43, HKU-1, NL63 and 229E respectively (Fig. 1c). These values were further extended by ‘hybrid’ infection-vaccine immunity with increments of 7070, 4.5, 4.2, 2.8 and 2.1 respectively (Fig. 1d, j).

These data show that strong humoral immunity against HCoV is seen across the life course with a distinct pattern of immunodominance. SARS-CoV-2 infection elicits a humoral response of moderate magnitude compared to that against HCoV, but this is extended 60-fold by primary series vaccination.

### SARS-CoV-2 and HCoV spike-specific titre correlate differentially at different stages of exposure to SARS-CoV-2

**Fig. 2:**
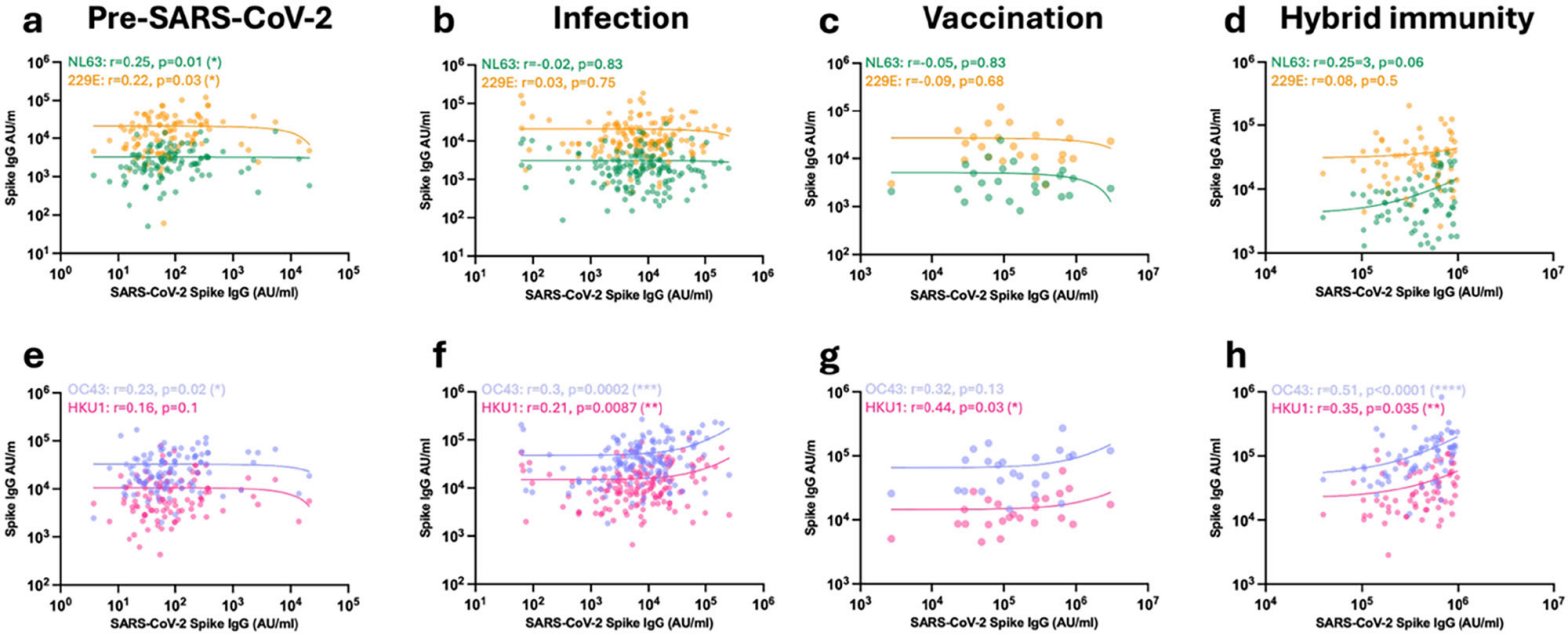
SARS-CoV-2 and HCoV spike-specific titre correlate differentially at different stages of exposure to SARS-CoV-2. Correlation of SARS-CoV-2 spike IgG titre with alphacoronavirus spike (NL63 (green) and 229E (yellow), **a**–**d**) and betacoronavirus spike (OC43 (purple) and HKU1 (pink), **e**–**h**) IgG titres in seronegative unvaccinated donors (**a**, *n* = 124), SARS-CoV-2-seropositive unvaccinated donors (**b**, *n* = 152), SARS-CoV-2-seronegative vaccinated donors (**c**, *n* = 24) and SARS-CoV-2-seropositive vaccinated donors (**d** *n* = 69). The Spearman correlation coefficient (*r*) and *P*-values are shown. Source data: Supplementary Data 1.

The correlation between SARS-CoV-2 and HCoV antibody titre within individual donors was next examined in relation to SARS-CoV-2 infection or vaccination status (Fig. 2).

Prior to SARS-CoV-2 infection or vaccination, the background antibody response against SARS-CoV-2 was correlated with antibody titres against all HCoVs except HKU1, indicating modest humoral cross-reactivity within the coronavirus family. Subsequent infection strongly increased this correlation with the betacoronaviruses, whilst that with alphacoronaviruses was lost. Of interest, no correlation was observed following vaccination in infection-naive donors, whilst this pattern was clearly established in donors with hybrid immunity.

These data indicate that antibody responses against most coronaviruses show some degree of cross-reactivity with SARS-CoV-2 prior to infection, whilst the correlation with antibodies against betacoronaviruses is strongly enhanced by infection but not vaccination.

### Antibodies against betacoronavirus HCoV can cross-react with the S2 domain of SARS-CoV-2

As spike-specific antibody titres against HCoV betacoronaviruses were enhanced preferentially compared to alphacoronaviruses following SARS-CoV-2 infection or vaccination, we next examined to what extent this increment was able to bind to SARS-CoV-2 spike or if it represented non-crossreactive ‘backboosting’. We were also interested to determine the relative importance of the S1 and S2 domains in cross-reactive recognition. To assess this, plasma from donors with prior SARS-CoV-2 infection was incubated with recombinant S1 or S2 SARS-CoV-2 spike domain protein. This pre-adsorbed plasma was then assessed for ability to bind to the spike domain of each HCoV.

**Fig. 3:**
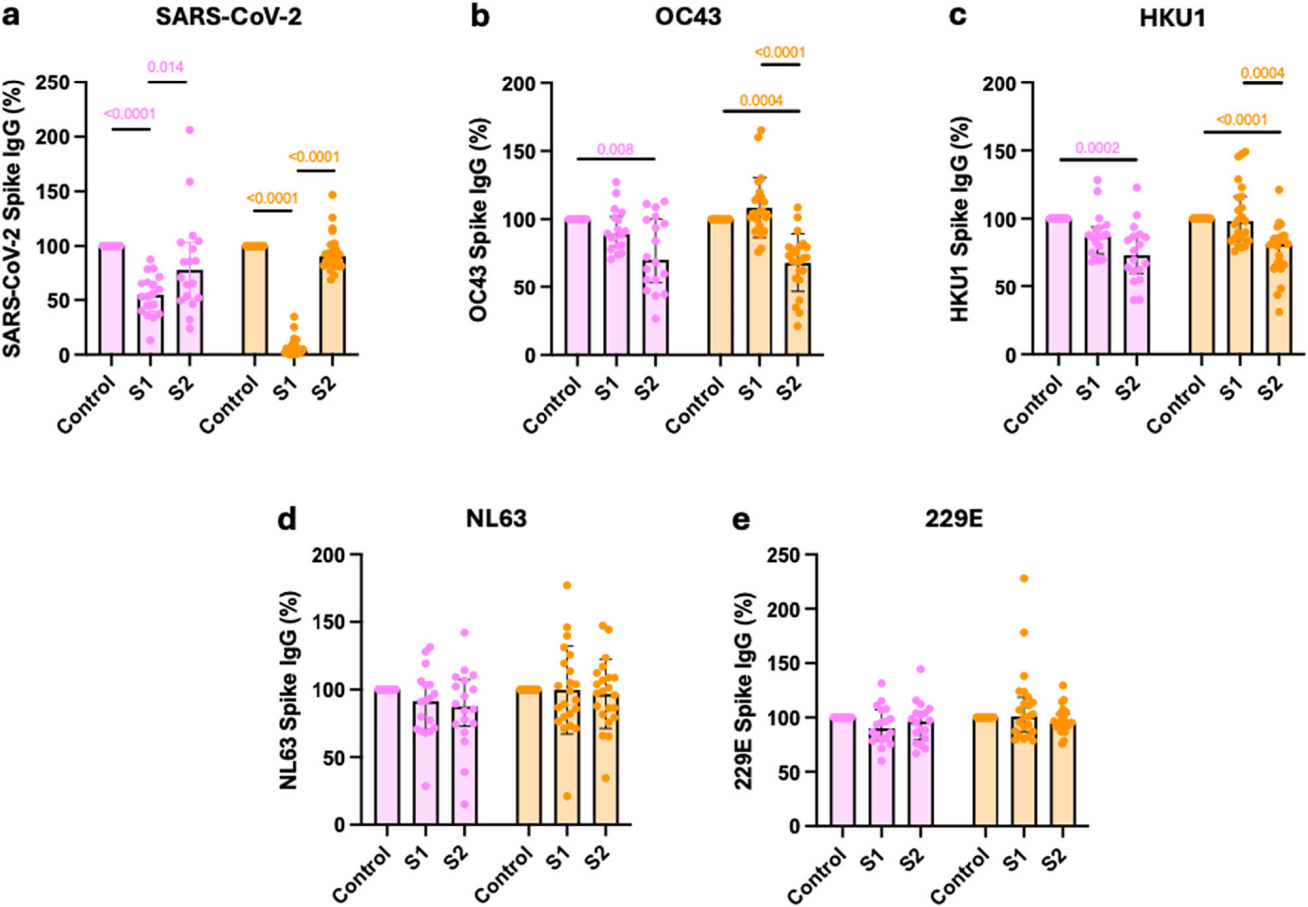
SARS-CoV-2 vaccination focusses antibody production against SARS-CoV-2 S1 domain whilst HCoV cross-reactive antibodies bind to the S2 domain. SARS-CoV-2 (**a**), OC43 (**b**), HKU1 (**c**), NL63 (**d**) and 229E (**e**) spike-specific IgG binding from unvaccinated (pink, *n* = 18) or vaccinated (orange, *n* = 23) SARS-CoV-2-seropositive donors following pre-adsorption with either S1 or S2 domains from SARS-CoV-2 Spike protein. Data are expressed as a percentage compared to binding within the unadsorbed control (Control). Friedman test with Dunn’s multiple comparison test was used. Source data: Supplementary Data 1.

As expected, antibody binding to SARS-CoV-2 spike was lost following adsorption with S1 and S2 domain. Preadsorption with S1 domain alone reduced total spike binding by 35% but this increased to 93% in vaccinated donors. As such, vaccination strongly biases the antibody response towards the S1 domain. (Fig. 3a, unvaccinated *p* = 0.0004, vaccinated *p* < 0.0001).

In contrast, the cross-reactive antibody response between SARS-CoV-2 and the OC43 and HKU1 HCoVs was largely focussed against the S2 domain (Fig. 3b, c).

Antibody binding to alphacoronaviruses NL63 or 229E was not reduced by SARS-CoV-2 spike preadsorption. As such, the increment in this titre that arises following SARS-CoV-2 vaccination or hybrid immunity must reflect backboosting of NL63 or 229E-selective B cells (34 d, 3e).

### Cellular responses against SARS-CoV-2 preferentially target S1 spike domain whilst HCoV-specific responses are largely S2 focussed

**Fig. 4:**
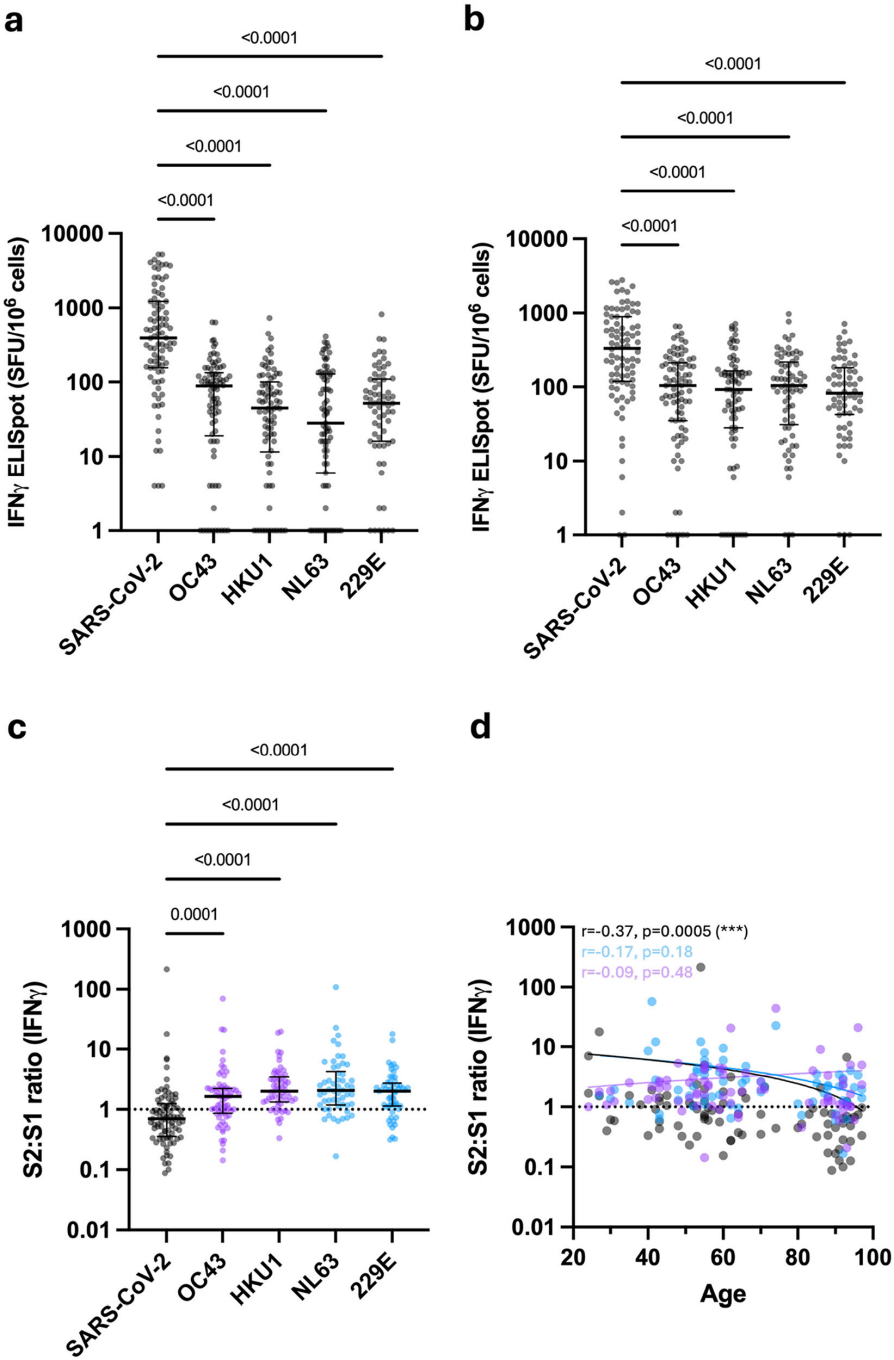
Cellular response to SARS-CoV-2 and HCoV measured by FluoroSpot. **a** IFNγ FluoroSpot response to SARS-CoV-2 and HCoV S1 peptide stimulation (*n* = 66–87). Kruskal–Wallis (Dunn’s multiple comparisons test). Black lines indicate the median and interquartile range. **b** IFNγ FluoroSpot response to SARS-CoV-2 and HCoV S2 peptide stimulation (*n* = 64–86). Kruskal–Wallis (Dunn’s multiple comparisons test). Black lines indicate the median and interquartile range. **c** Ratio of S2-specific to S1-specific IFNγ cellular responses in SARS-CoV-2 and HCoV (*n* = 54–84). Betacoronaviruses are indicated with purple dots, and alphacoronaviruses are indicated with blue dots. Kruskal–Wallis (Dunn’s multiple comparisons test). Black lines indicate the median and interquartile range. **d** Ratio of S2-specific to S1-specific IFNγ cellular responses to SARS-CoV-2 (black dots, *n* = 84), alphacoronaviruses NL63 and 229E (blue dots, *n* = 66) and betacoronaviruses OC43 and HKU1 (purple dots, *n* = 74) in relation to age. Dotted black line indicates a S2:S1 ratio of 1. The Spearman correlation coefficient and *P*-values are shown. Fitted lines are linear regressions. Source data: Supplementary Data 1.

We next went on to determine the relative magnitude and specificity of the cellular response to spike peptides from SARS-CoV-2 and HCoV. PBMC were isolated from donors with prior SARS-CoV-2 vaccination and assessed in a quantitative IFNγ and IL-2 FluoroSpot assay utilising overlapping peptide pools from the S1 and S2 domains of the spike protein from SARS-CoV-2, OC43, HKU1, NL63 or 229E (Fig. 4).

Spike-specific cellular responses were substantially higher against SARS-CoV-2 compared to HCoV, regardless of SARS-CoV-2 infection status, and broadly comparable against S1 and S2 domains (Fig. 4a, b, *p* < 0.0001). In contrast, a discordant pattern of relative immunity against S1 and S2 peptides was observed for HCoVs. Relative immunodominance against HCoV S1 was comparable to that observed for humoral immunity, with OC43 > 229E > HKU1 > NL63. However, cellular responses against peptides from S2 were broadly comparable across all HCoV. To assess this profile further, cellular responses to peptides from S2 or S1 were expressed as a ratio. The median S2:S1 ratio was 0.69 for SARS-CoV-2 but increased to 1.6, 2.0, 2.1 and 2 for OC43, HKU1, NL63 and 229E, respectively (Fig. 4c, *p* = 0.0001- < 0.0001, Supplementary Fig. 3c). S2:S1 ratio in relation to age showed that older people have comparatively stronger cellular responses to S1 peptides from SARS-CoV-2 (Fig. 4d, *r* = −0.37, *p* = 0.0005). No significant trends pertaining to age were observed for HCoV.

These findings demonstrate that cellular responses to SARS-CoV-2 preferentially target the S1 spike domain following vaccination, whilst cellular responses to HCoV are focussed against S2.

### T cell recognition of human betacoronaviruses elicits a common cytokine profile of IFNγ, IL-2 and CXCL8 production

**Fig. 5:**
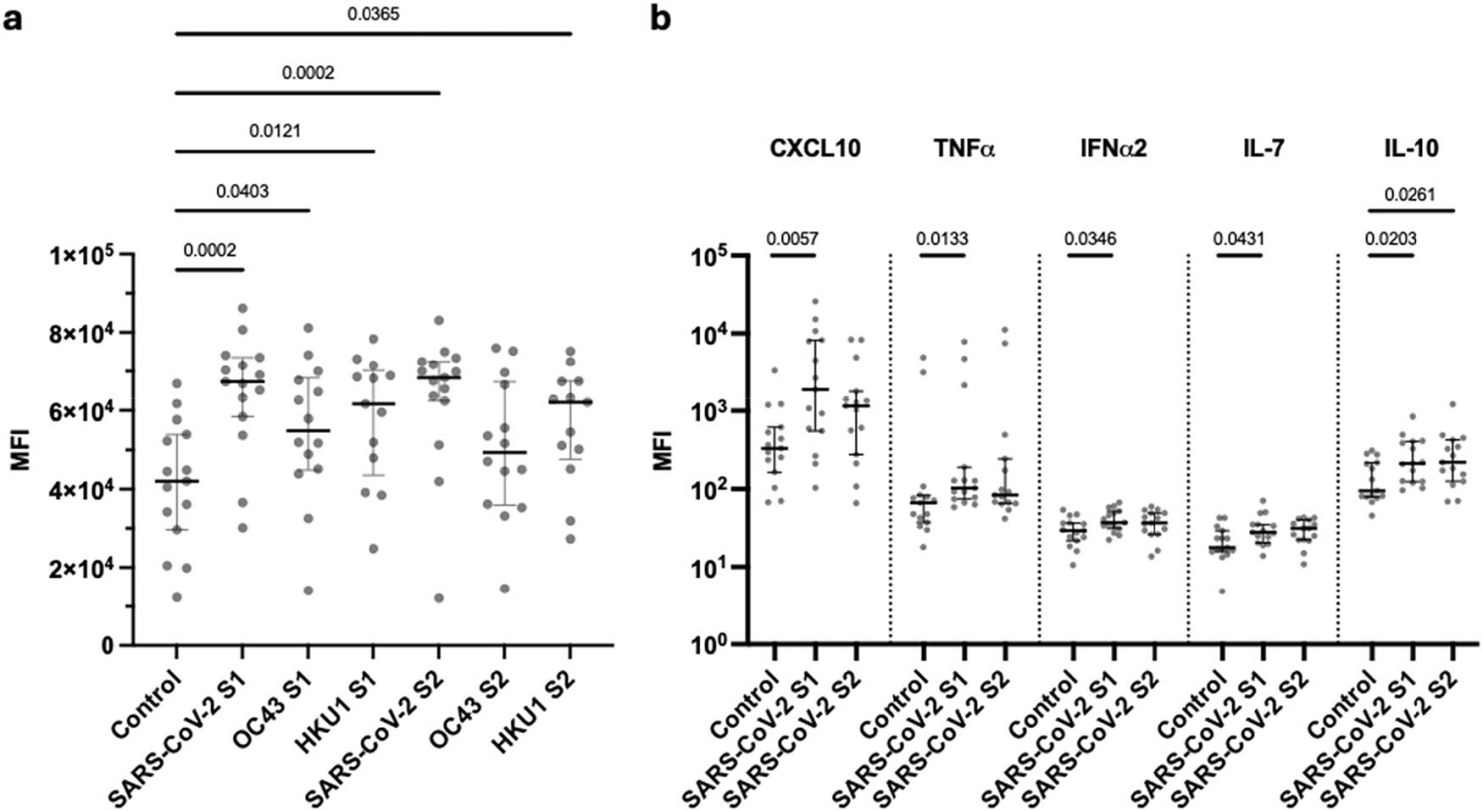
Profile of cytokine production following cellular recognition of spike peptides from betacoronaviruses. **a** Mean fluorescence intensity (MFI) of CXCL8 within FluoroSpot eluates after stimulation with either S1 or S2 spike peptides from SARS-CoV-2 or OC43 and HKU1 compared to DMSO control. Donors were SARS-CoV-2 vaccinated donors and >65 years of age (*n* = 15). Kruskal–Wallis (uncorrected Dunn’s test). Black lines indicate the median MFI with interquartile range. **b** Mean fluorescence intensity (MFI) of CXCL10, TNFɑ, IFN-ɑ2, IL-7 and IL-10 within FluoroSpot eluates after stimulation with SARS-CoV-2 S1 or S2 peptides compared to a DMSO control in vaccinated donors over the age of 65 (*n* = 15). Kruskal–Wallis (uncorrected Dunn’s test). Black lines indicate the median MFI with interquartile range. Source data: Supplementary Data 2.

The functional capacity of coronavirus-specific T cells was further assessed by analysis of the profile of cytokine production following peptide stimulation. IFNγ and IL-2 production had been determined by FluoroSpot, and this was extended using LEGENDplex analysis of eluates from FluoroSpot plates following spike peptide stimulation. Given the importance of age as a risk factor for coronavirus infection, these assays were undertaken in donors aged over 65 years with prior SARS-CoV-2 infection (Fig. 5; Supplementary Data 2).

A striking observation was that concentrations of CXCL8 were markedly increased following stimulation with S1 or S2 spike peptides from betacoronaviruses (Fig. 5a). CXCL10, TNFɑ, IFNɑ2, IL-7 and IL-10 were also elevated following SARS-CoV-2 S1 stimulation, whilst IL-10 increased following stimulation with peptides from S2. (Fig. 5b). These cytokines were not elevated following recognition of peptides from HCoV (Supplementary Fig. 4).

## Discussion

SARS-CoV-2 is now established as the 5th endemic coronavirus and the magnitude and profile of heterologous immune recognition within this taxonomic group will impact directly on the future clinical burden from coronaviruses. Our findings document the profile of humoral and cellular immune recognition of the spike protein from each of the 5 coronaviruses during the emergence of SARS-CoV-2 and reveal only a modest impact on HCoV-specific immunity. These observations provide insights into the mechanisms and potential future clinical importance of coronavirus-specific immune recognition.

Previous studies have shown little cross-reactivity of pre-pandemic HCoV-specific antibodies to bind to SARS-CoV-2 spike protein and this was observed in our study. The structural evolution of SARS-CoV-2 away from recognition by prior betacoronavirus-specific antibody binding is likely to have been a critical factor in its high infectious capacity. Nevertheless, cross-reactive antibodies of low to moderate titre were seen in 10% of donors, in line with previous reports^21^. This has previously been reported in children, although in our study these antibodies were more common in older donors. The reason for this is not clear but may potentially reflect more recent HCoV infection due to immune senescence. Cross-reactive antibody titre correlated with titres against all HCoV except HKU1 and it is likely that these bind to spike S2 domain and are of modest clinical importance. Additional features prior to SARS-CoV-2 were that virtually all donors had been infected with each of the HCoV and that an established immunodominance was apparent across the population with the strongest antibody titres against OC43 and 229E. The mechanisms behind this latter observation are unclear but could represent a relative response to repeated infections^22^.

SARS-CoV-2 infection reliably elicited a virus-specific antibody response, but it was notable that this was of moderate titre compared to HCoV and remained lower than OC43 or 229E. Again, this may reflect a relatively attenuated humoral response to a primary coronavirus infection as titre increases with repeat infection^23^. Infection also selectively, and modestly, boosted antibody titre against the betacoronaviruses by <2-fold. SARS-CoV-2 vaccination increased this effect and also evoked a small increase in cross-reactive response against alphacoronaviruses^24^. A particularly striking increase in antibody response was apparent in donors with a hybrid immune status of prior infection and vaccination, with >4-fold and >2-fold increase in titre against beta and alphacoronaviruses, respectively. The mechanisms that underlie this synergistic response are uncertain but may not be maintained given the propensity for antibody waning following SARS-CoV-2 vaccination^25^.

Analysis of these trends across the adult life course showed relatively little impact of age, although SARS-CoV-2-specific antibody response in hybrid immunity was higher in older people. Strong vaccine-induced immunity has been shown in LTCF recipients, and a potential confounding factor is that these assays include only those donors who were able to survive SARS-CoV-2 infection^17^. Overall, despite this moderate boosting of coronavirus-specific antibody titre following SARS-CoV-2 infection and/or vaccination, it was notable that the pattern of HCoV immunodominance remained unaltered.

Correlation of HCoV-specific antibody titre with antibody binding to SARS-CoV-2 spike revealed a number of notable observations. As discussed above, higher levels of antibody to each HCoV, except HKU1, correlated with background levels of SARS-CoV-2 binding in pre-immune donors. This relationship was further enhanced for betacoronaviruses after infection, but it was notable that the SARS-CoV-2-specific antibody response following vaccination alone, in the absence of prior infection, was not related to HCoV-specific titre and indicates that vaccination drives a somewhat different form of spike-specific humoral response compared to infection. To investigate these patterns further, the relative importance of the two Spike protein domains in cross-reactive antibody responses was next determined through pre-adsorption of SARS-CoV-2 immune serum with S1 or S2 protein domains. As expected, this assay substantially depleted antibody binding against SARS-CoV-2 spike, although the relative specificity of the humoral response was markedly impacted by vaccination. In particular, whilst S1-specific antibodies were preferentially increased after infection, this pattern was strongly enhanced after vaccination with relatively negligible residual S2-specific response^26^. It was also notable that preadsorption with the SARS-CoV-2 S1 domain led to an increase in binding against HCoV in some donors. The cause of this is unclear, but may potentially indicate the presence of inhibitory cross-reactive antibodies.

Amino acid sequence conservation within HCoV is higher within the spike S2 domain, and it was notable that cross-reactive antibodies against betacoronaviruses were largely directed against the SARS-CoV-2 S2 domain, in line with prior reports^27–29^. Of interest, pre-adsorption studies showed that the increased antibody response against alphacoronaviruses NL63 and 229E that was observed after SARS-CoV-2 vaccination was not cross-reactive against the SARS-CoV-2 spike protein. As such, this likely reflects indirect ‘back-boosting’ of NL63 and 229E-specific B cell clones through very low-affinity antibody engagement with SARS-CoV-2 spike and/or CD4+ follicular T cell help. A non-specific bystander activation of HCoV-specific B cells may also contribute.

Analysis of T cell immune responses in SARS-CoV-2 vaccinees revealed a strong SARS-CoV-2-specific response, which was relatively evenly directed against peptides from the S1 and S2 domains. It was notable that the magnitude of the SARS-CoV-2-specific response was markedly greater than cellular recognition of HCoVs, indicating that, despite the relative tolerance of T cell recognition for amino acid variation, the SARS-CoV-2-specific T cell response was largely non-cross-reactive with HCoV peptides. A different pattern was observed for cellular responses to HCoV spike peptides. These were preferentially focussed against the S2 domain and were comparable in magnitude between the 4 viruses, potentially indicating selection for cross-reactive T cells over time. Indeed, a trend was observed towards increased recognition of S2 peptides with aging and this would be of interest to study in future work. Also of interest, the pattern of immunodominance of S1-specific peptide recognition between HCoV demonstrated the same profile that was observed for antibody response, potentially indicating a preferential role for S1-specific recognition by T cells in supporting B cell development. No impact of age was observed in relation to the magnitude of T cell recognition, although low levels of T cell response to betacoronaviruses have been reported in pre-pandemic samples^30^. A further observation was for a relative focus of SARS-CoV-2-directed T cell immunity against the S1 domain in older people, a pattern not seen for alpha or betacoronaviruses. The underlying basis for this is not clear, but may potentially reflect the selection of elderly donors who survived primary SARS-CoV-2 infection or a robust response to recent vaccination.

T cell recognition of coronavirus peptides elicited release of IFNγ and IL-2, and extended cytokine analysis also revealed high levels of CXCL8 following stimulation with betacoronavirus spike peptides. CXCL8 chemokine is a potent attractant for neutrophils and heightened levels correlate with COVID-19 severity^31^. CXCL8 is a dominant cytokine produced from T cells in infancy, and this may potentially indicate an adaptive role in control of coronavirus infection early in life^32^. The potential role of CXCL8 in the control of betacoronavirus infection is unclear, but could include support for local inflammatory response with anatomical limitation of virus transmission and promotion of airway clearing.

This study has potential limitations, including the fact that LTCF residents have increased frailty compared with community-dwelling age-matched peers, as well as potential selection bias for older donors who were survivors of primary SARS-CoV-2 infection. We also did not have access to the date or severity of SARS-CoV-2 infection, and the prevalence of HCoV infection during this period was not monitored within the community. It would also be of interest to assess neutralising antibody activity in addition to Spike-specific titre. Furthermore, our work only addressed cross-reactive immunity against the spike protein, and heterologous recognition of more conserved regions of the viral genome may be of value following SARS-CoV-2 infection^33^.

In conclusion, adaptive immune responses to the spike protein of HCoV demonstrate a characteristic virus-specific pattern of immunodominance in humoral and S1-specific cellular recognition with comparable cellular recognition of S2 across all viruses. SARS-CoV-2 infection impacts marginally on this profile, although hybrid immunity enhances antibody responses due to cross-reactive and back-boosted humoral immunity. However, these responses are modest in magnitude and, given the importance of spike protein in mediating HCoV tropism, there is little to indicate that markedly increased immune protection against HCoV infection has developed within the population following SARS-CoV-2 evolution. Further, whilst vaccination drives strong antibody responses against S1 domain, thereby potentially enhancing neutralising responses, the pattern of S2-focussed cellular responses against HCoV may indicate a transition towards recognition of the SARS-CoV-2 S2 domain over time. Given the relative conservation of amino acid sequence within S2 this transition has the potential to provide robust immune recognition of novel SARS-CoV-2 variants where mutations are typically focussed within the S1 domain. Overall, these findings suggest that post-pandemic HCoV infection rates may continue at similar levels as seen prior to 2020 and the development of a pan-coronavirus-specific vaccine is thus a priority.

## Supplementary information

Supplementary Information

Description of Additional Supplementary Files

Supplementary Data 1

Supplementary Data 2

Reporting Summary

## Data Availability

De-identified test results and limited metadata will be made available for use by researchers in future studies, subject to appropriate research ethical approval. Source data can be requested from the corresponding author. Source data for Table 1 and Figs. 1–4 are found in ‘Supplementary Data 1 for Figs. 1–4’. Source data for Fig. 5 is in ‘Supplementary Data 2 for Fig. 5’.
