# Supplementary material for: Endemic penetrance of SARS-CoV-2 has impacted marginally on immunity to spike protein of human coronaviruses
Source: Commun Biol. 2026 Jan 3;9:196. doi: 10.1038/s42003-025-09474-x (PMC12886799; doi:10.1038/s42003-025-09474-x)
Supplement: Supplementary file 1 — Supplementary Information [file 42003_2025_9474_MOESM1_ESM.pdf]

Supplementary figures

S1

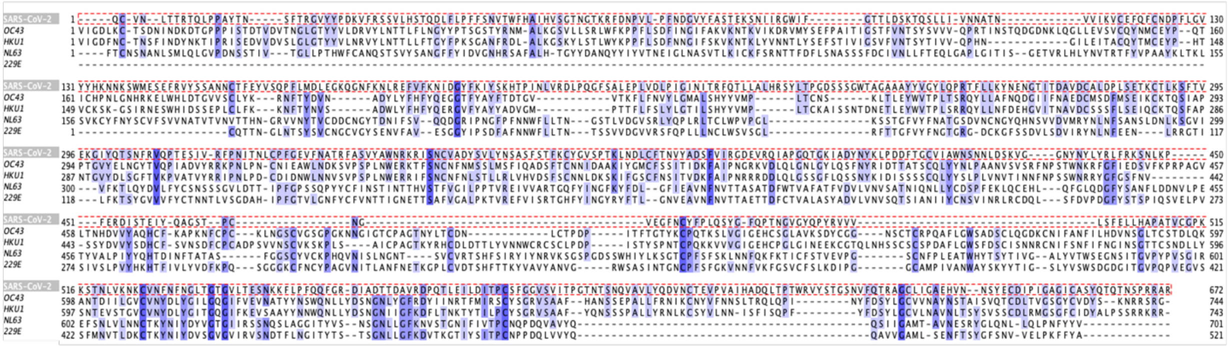

S2

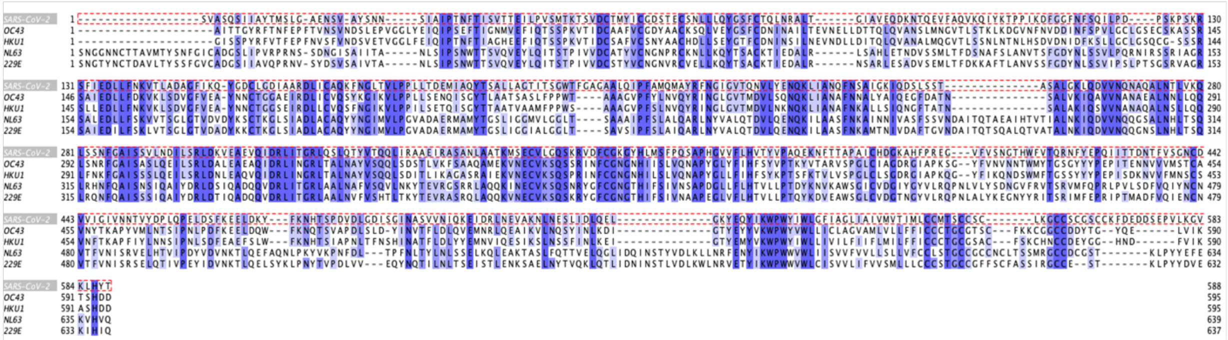

	Homology S1 (%)	Homology S2 (%)
SARS-CoV-2	100	100
OC43	30	42
HKU1	31	41
NL63	29	34
229E	28	35

Supplementary Figure 1: Multiple alignment of S1 and S2 domains of Spike protein from SARS-CoV-2 and four endemic human coronaviruses

Alignment created using NCBI Cobalt. Darker blue indicates regions of high homology. Table contains the percentage identity of four endemic coronaviruses (OC43, HKU1, NL63 and 229E) in relation to SARS-CoV-2 (Wuhan strain) from NCBI blastp.

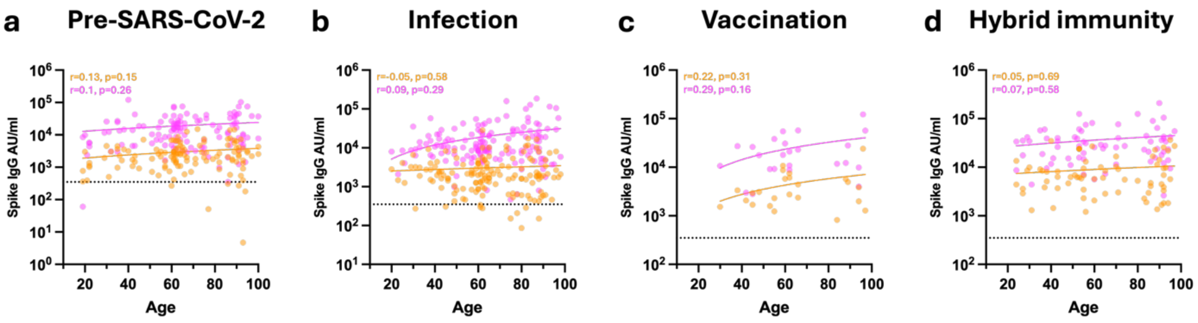

Supplementary Figure 2: Alphacoronavirus spike IgG titres do not correlate with donor age
Correlation of NL63 (orange) and 229E (pink) spike IgG titres with age in seronegative unvaccinated (a, n=124), seropositive unvaccinated (b, n=152), seronegative vaccinated (c, n=24) and seropositive vaccinated (d, n=69) donors. The Spearman correlation coefficient (R) and P-values are shown.

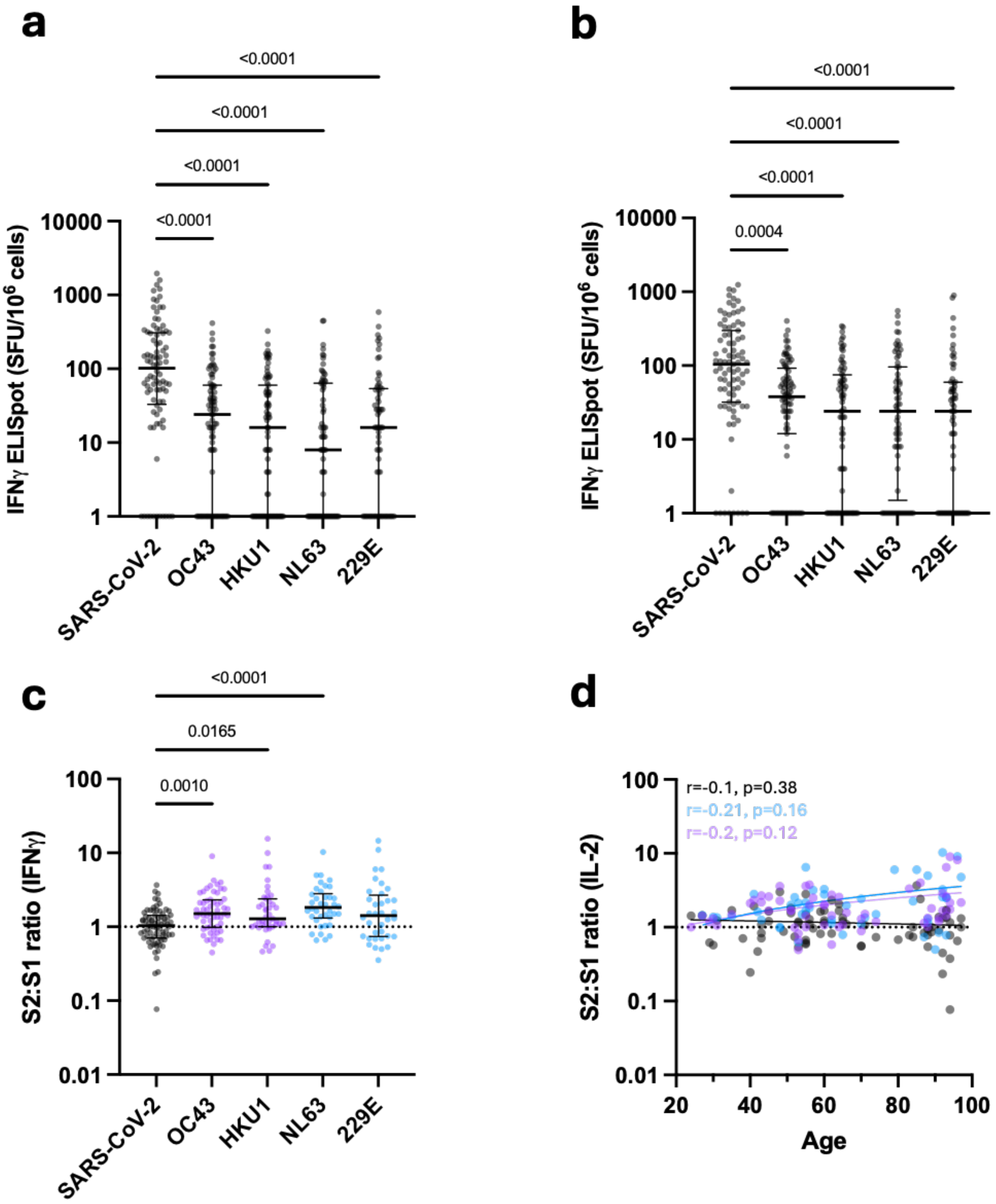

Supplementary Figure 3: IL-2 Cellular response to SARS-CoV-2 and endemic coronaviruses OC43, HKU1, NL63 and 229E measured by FluoroSpot

a. IL-2 FluoroSpot response to SARS-CoV-2 and endemic coronavirus S1 peptide stimulation (n=67-88). Kruskal-Wallis (Dunn's multiple comparisons test). Black lines indicate the median and interquartile range.

b. IL-2 FluoroSpot response to SARS-CoV-2 and endemic coronavirus S2 peptide stimulation (n=64-87). Kruskal-Wallis (Dunn's multiple comparisons test). Black lines indicate the median and interquartile range.

c. Ratio of S2-specific to S1-specific IL-2 cellular responses in SARS-CoV-2 and endemic human coronaviruses (n=36-74). Betacoronaviruses are indicated with purple dots, and alphacoronaviruses are indicated with blue dots. Kruskal-Wallis (Dunn's multiple comparisons test). Black lines indicate the median and interquartile range.

d. Ratio of S2-specific to S1-specific IFNγ cellular responses to SARS-CoV-2 (black dots, n=74), alphacoronaviruses NL63 and 229E (blue dots, n=46) and betacoronaviruses OC43 and HKU1 (purple dots, n=58) in relation to age. Dotted black line indicates a S2:S1 ratio of 1. The Spearman correlation coefficient (R) and P-values are shown. Fitted lines are linear regressions.

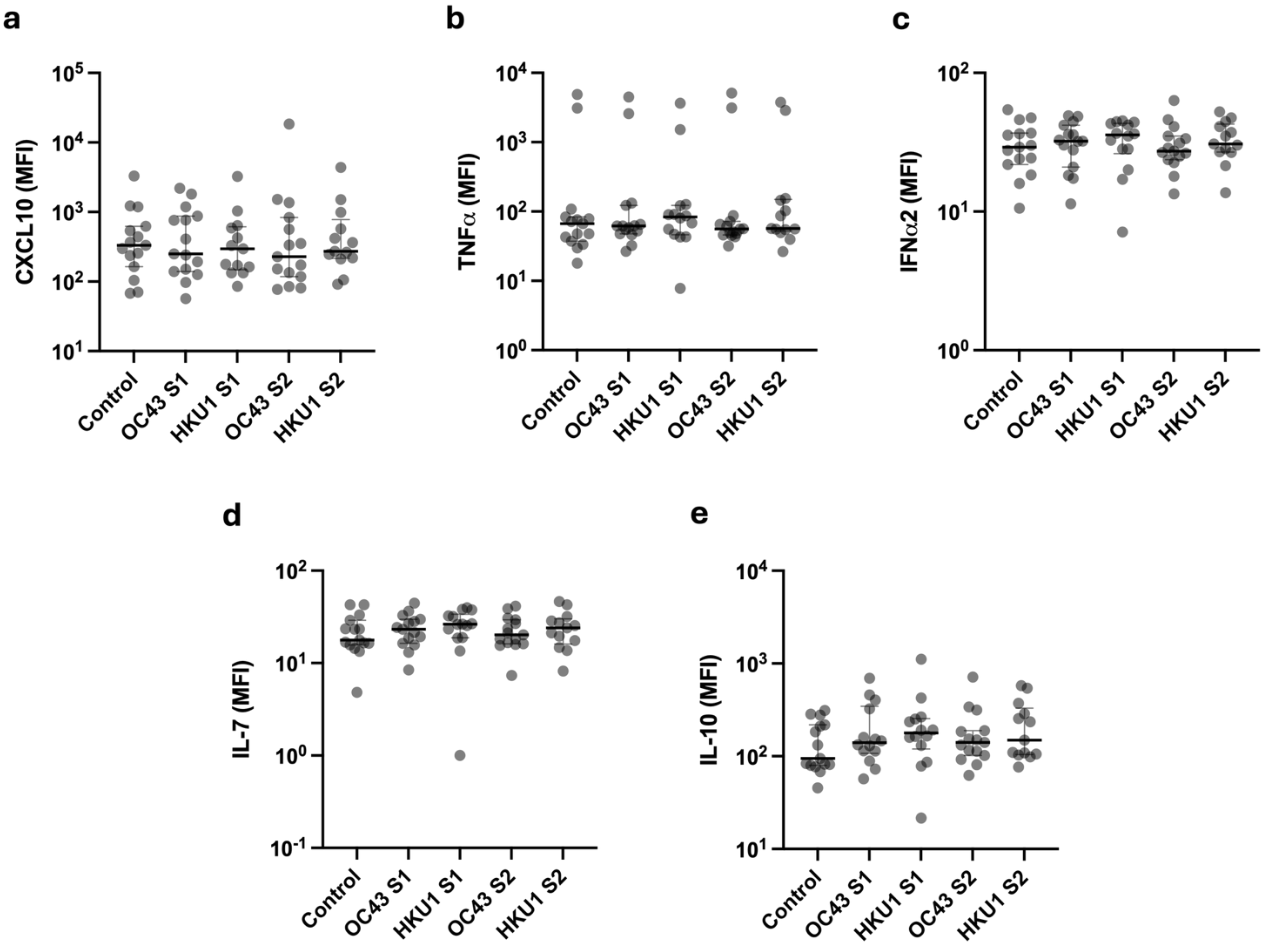

Supplementary Figure 4: Pro-inflammatory cytokine production in SARS-CoV-2 seropositive donors over the age of 65 following stimulation with spike peptides from betacoronaviruses
Mean fluorescence intensity (MFI) of CXCL10 (A), TNFα (B), IFNα2 (C), IL-7 (D) and IL-10 (E) in FluoroSpot eluates after stimulation with OC43 spike S1 (dark green), HKU1 spike S1 (dark blue), OC43 spike S2 (light green), or HKU1 spike S2 (light blue) peptides from betacoronaviruses compared to a DMSO control (red) in vaccinated donors over the age of 65 (n=15). Kruskal-Wallis (uncorrected Dunn's test). Black lines indicate the median MFI with interquartile range.
