# Supplementary material for: Endemic penetrance of SARS-CoV-2 has impacted marginally on immunity to spike protein of human coronaviruses
Source: Commun Biol. 2026 Jan 3;9:196. doi: 10.1038/s42003-025-09474-x (PMC12886799; doi:10.1038/s42003-025-09474-x)
Supplement: Supplementary file 2 — Description of Additional Supplementary Files [file 42003_2025_9474_MOESM2_ESM.pdf]

Description of Additional Supplementary Files

File name: Supplementary Data 1 for Figs 1-4.xlsx

Description: Data set of antibody and cellular responses for Table 1 and Figures 1-4

File name: Supplementary Data 2 for Fig 5.xlsx

Description: Data set of cytokine concentration in LEGENDPlex assay for Figure 5
